# Orbital varix: rare cause of blepharospasm

**DOI:** 10.11604/pamj.2019.32.147.14958

**Published:** 2019-03-26

**Authors:** Badii Hmida, Walid Mnari, Mezri Maatouk, Ahmed Zrig, Mondher Golli

**Affiliations:** 1 Imaging Department, FB University, Hospital Monastir, Tunisia

**Keywords:** Orbit, varix, CT scan, MRI

## Abstract

Orbital varix (or varicose) is an exceptional pathology with poor clinical sign. The blepharospasm can be a revealing cause. The long-term risk is optic atrophy and blindness. Magnetic resonance imaging is the best diagnostic tools. The rise of lesion dimensions by Valsalva maneuvers and prone position is characteristic. We report the observation of a 42-year-old young man, consulting for a blepharospasm of the left eye evolving for two years. Imaging investigations made the diagnosis of orbital varicose.

## Introduction

The orbital varix are dilations and proliferations of intraorbital venules [1]. They can be congenital or secondary. They represent less than 2% of the orbital tumors [2]. The clinical signs in touch with the uncomplicated orbital varix veins are few mainly intermittent and positional exophthalmitis, often painful, or a blepharospasm.

## Patient and observation

**Figure 1:**
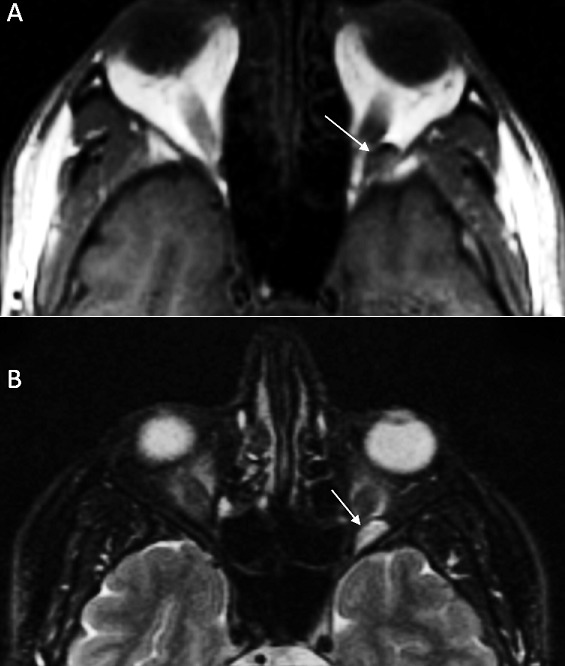
axial images of orbital MRI showing a left intra-orbital process which is low signal in T1 (A) and high signal in T2 (B)

**Figure 2:**
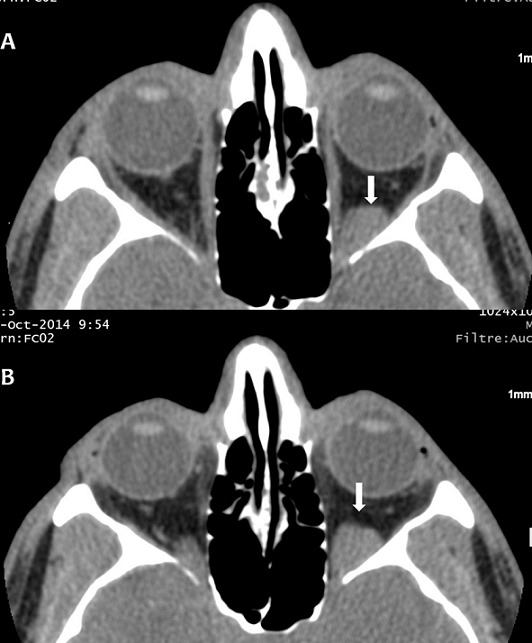
orbital CT in spontaneous contrast showing a left retro-orbital mass (A, arrow); note the volume increase of the mass in prone position (B, arrow)

A 42-year-old young man, a plumber, without notable pathological antecedents, consulting for a blepharospasm of the left eye evolving for two years. The patient had no clinically significant ocular history. There is no notion of trauma or accidental introduction of intraocular foreign body. The neurological examination was normal. There is a blepharospasm without proptosis. The patient's visual acuity was 20/20 in the two eye. Visual fields were intact, pupillary responses were equal and indirect fundoscopy was normal. All biochemical and hematological indices were normal. The cerebral MRI was unremarkable. The axial fine sections passing through the orbits show a left retro-orbital process in T1 low signal and T2 hyper signal (Figure 1), weakly enhanced after Gadolinium injection. There is no phlebolite or sign of thrombosis. The vascular nature of the retro-orbital lesion was evoked and confirmed by orbital CT scan. In the prone position, the CT showed an increase in the volume of this process (Figure 2). The Doppler Ultrasonogrphy confirmed these findings by showing a retro-orbital varicose vein that increases in volume at Valsalva maneuver and the sitting position tilted forward. Those imaging findings are compatible with the diagnosis of non-thrombotic orbital varix. The patient was treated with botulinum toxin injection for blepharospasm, without improvement. The patient refused endovascular treatment.

## Discussion

The orbital varicose is rare. They are a dilation of venules developing most of the time from the ophthalmic vein [1, 2]. They are thin walled, low flow and distensible vessels. The etiology of orbital varicosities is unknown and the literature is sparse. They are most commonly asymptomatic. In absence of complication, varicose is clinically characterized by an intermittent exophtalmos exacerbated by the Valsalva maneuver; this increases pressure in intra orbital vein and majors the dilation of the vessels. Rarely does it cause a blepharospasm or an enophthalmos. These symptoms may be explained by the pressure and conflict of the huge vascular formations with oculomotor muscles [1-3]. Magnetic resonance imaging is the best diagnostic tools for orbital varicose. It is the first imaging examination who must be done for orbital clinical signs. The MRI signal is variable due to fast or relented flow in dilated veins. The varix can appear as high signal T2 weighted images - low T1 weighted images and so can mimic tissue lesions. Gadolinium injection sequences are essential to search thromboembolic complications [1, 4, 5].

Doppler Ultrasonography confirms the vascular nature of the lesion [1, 4]. It can shows also an increase of the size as well as hemodynamic variations during the Valsalva maneuver or similar action (eg, coughing). The increase in the size of the lesions during the prone position is easier to done on CT scan. It can also show phlebolite, search for thrombosis or signs of hemorrhagic complications [6]. However, orbital venography still the key examination for positive diagnosis, but it currently used us the first time of an embolization treatment [3]. The differential diagnosis of an orbital varicose includes other causes of orbital masses such us lymphoma, metastatic tumors, and inflammatory nodes [6]. Treatment of ophthalmic varix by endovascular embolization associated or not with surgical excision is indicated for complicated forms mainly thrombosis or bleeding [1, 5]. In the absence of treatment, the long term-risk is optic atrophy and blindness [1, 4, 5].

## Conclusion

Orbital varicose veins are exceptional. Blepharospasm may be an evocative symptom. Radiological examinations, mainly MRI, are essential to showing the vascular nature of the orbital lesion. Prone maneuver, which increases the size of the varicose, can establish the positive diagnosis.

## Competing interests

The authors declare no competing interests.
